# Intravertebral collateral enhancement resembling sclerotic metastatic disease in a case of cervical epidural abscess

**DOI:** 10.1007/s00256-024-04761-w

**Published:** 2024-07-30

**Authors:** Daniel K. Schneider, Ahmet Hakan Ok, Claus S. Simpfendorfer, Michael C. Forney, Naveen Subhas

**Affiliations:** Imaging Institute, Cleveland Clinic, 9500 Euclid Ave, A21, Cleveland, OH 44195 USA

**Keywords:** Vertebral body enhancement, Pseudolesion, Sclerotic metastatic disease, Cervical epidural abscess, Central venous obstruction

## Abstract

Vertebral body enhancement is occasionally seen on postcontrast CT imaging in the absence of osseous pathology. This enhancement can mimic sclerotic osseous metastatic disease, leading to a diagnostic dilemma for radiologists and increasing the chance of misinterpretation. Existing literature has focused on the association between this enhancement and concomitant central venous system obstruction. We report a 61-year-old woman with a history of nasopharyngeal carcinoma presenting with an epidural abscess who exhibited vertebral body enhancement resembling sclerotic metastatic disease without imaging evidence of central venous obstruction or vertebral osseous metastatic disease. Awareness of this unique presentation may prevent the incorrect diagnostic errors and their associated negative effects on patients.

## Introduction

Sclerotic bone lesions are common and can have various etiologies, including primary neoplasms, infections, inflammatory conditions, and vascular anomalies. For example, reactive sclerosis and periosteal reaction may be seen in the setting of subacute and chronic osteomyelitis, particularly around the periphery of the infected bone. Many sclerotic primary neoplasms are benign, with common examples including bone islands (i.e., enostoses), osteomas, and healed non-ossifying fibromas. In patients with a history of cancer, these sclerotic lesions are more likely to be the result of metastatic malignancy [1]. The skeletal system is the third most common site to be involved in metastases, and the spine is the skeletal area most frequently affected by metastatic diseases [2]. While osteoblastic metastases are most frequently seen in patients with a history of prostate and breast cancer, they are not infrequently seen in patients with other primary malignancies such as nasopharyngeal carcinoma, which has an overall incidence of skeletal metastasis (both osteolytic and osteoblastic) of up to 15% [3, 4].

Vertebral body enhancement is occasionally seen on postcontrast CT imaging in patients without underlying osseous abnormalities. This phenomenon has been reported most frequently in the setting of central venous system obstruction. This enhancement poses a challenge for radiologists, who may consider sclerotic osseous metastases as a leading diagnosis in the absence of noncontrast imaging for comparison [5–9].

We present the case of a patient with an epidural abscess who exhibited vertebral body enhancement, resembling sclerotic metastatic disease, without central venous obstruction or malignancy but rather in the setting of upper cervical epidural abscess.

## Case report

**Fig. 1:**
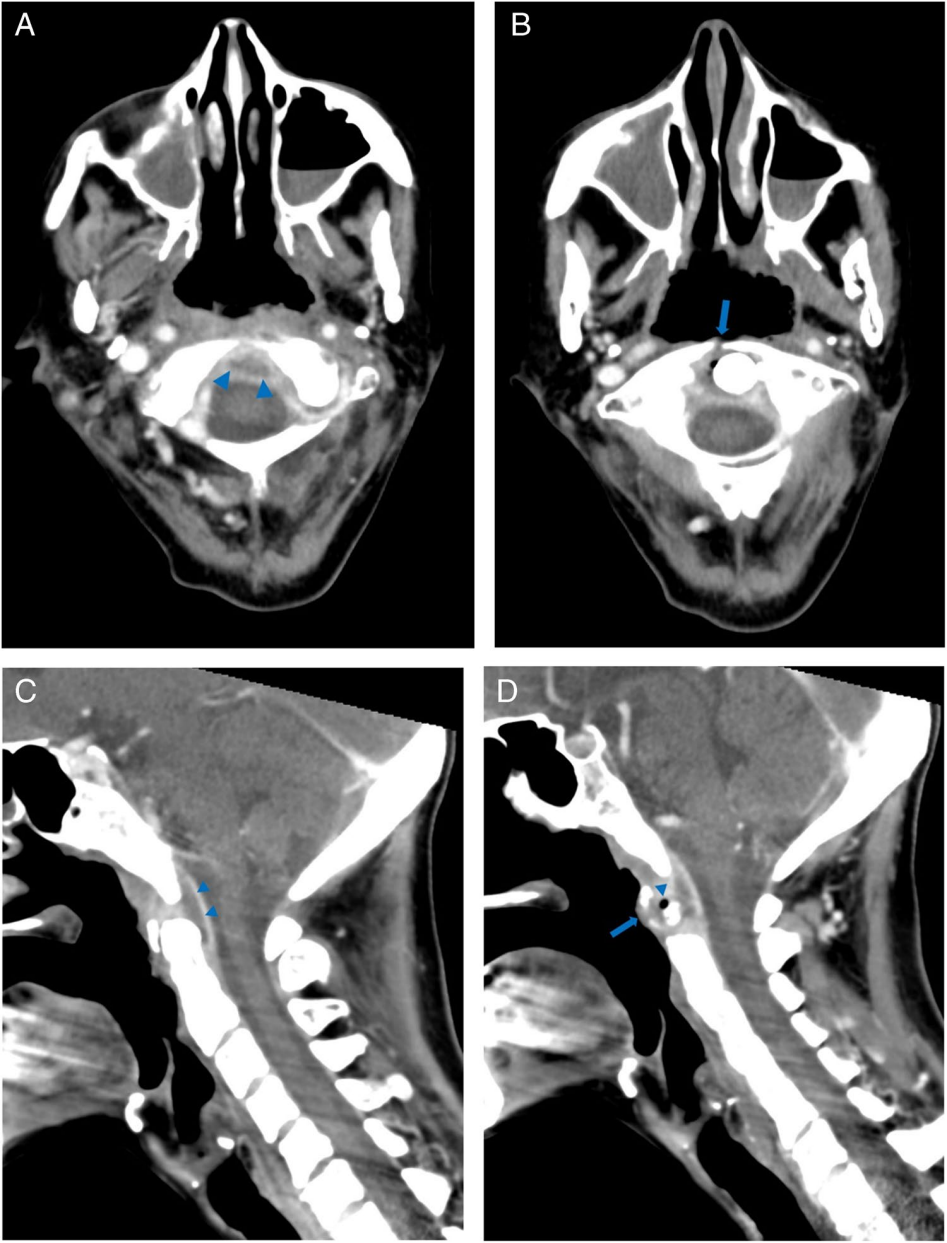
Sequential axial (**A** and **B**), midline (**C**), and paramidline (**D**) sagittal images from initial contrast-enhanced neck CT demonstrate dehiscence of the posterior oropharyngeal wall with underlying erosion in the C1 anterior arch (arrows) and peripherally enhancing gas-containing fluid collection surrounding the odontoid process (arrowheads)

**Fig. 2:**
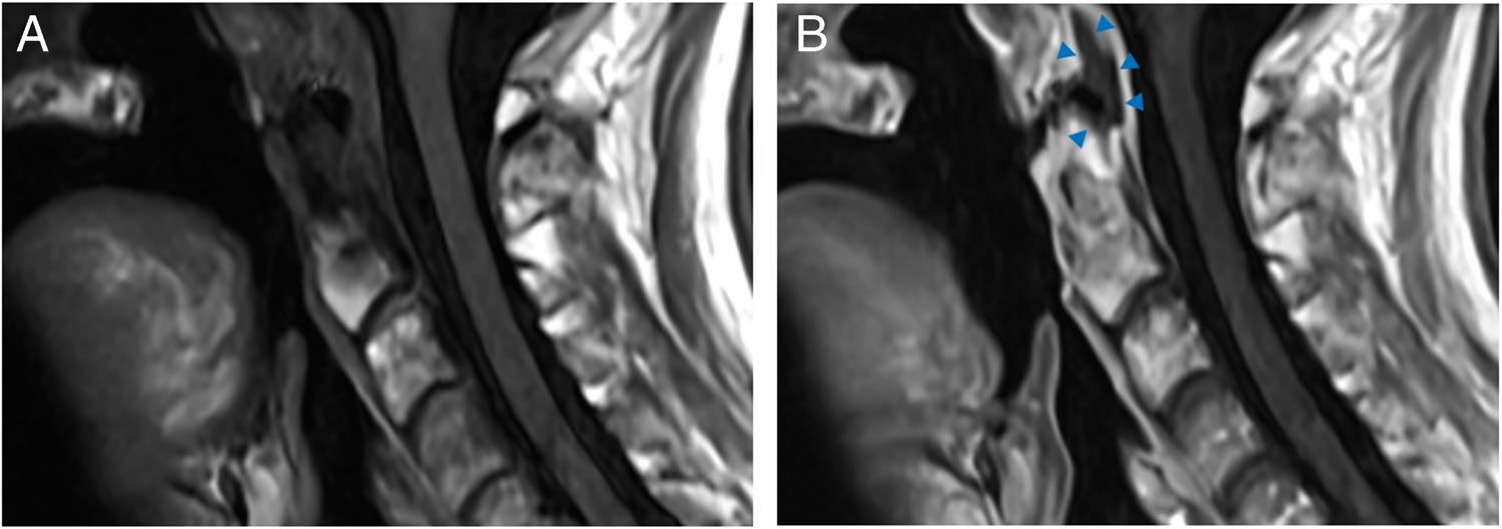
T1-weighted precontrast MR (**A**) and postcontrast MR (**B**) images demonstrate a peripherally enhancing fluid collection surrounding the odontoid process (arrowheads) with internal susceptibility corresponding to gas seen on CT. Cortical erosions, T1 hypointensity, and enhancement in the odontoid process are consistent with acute osteomyelitis

**Fig. 3:**
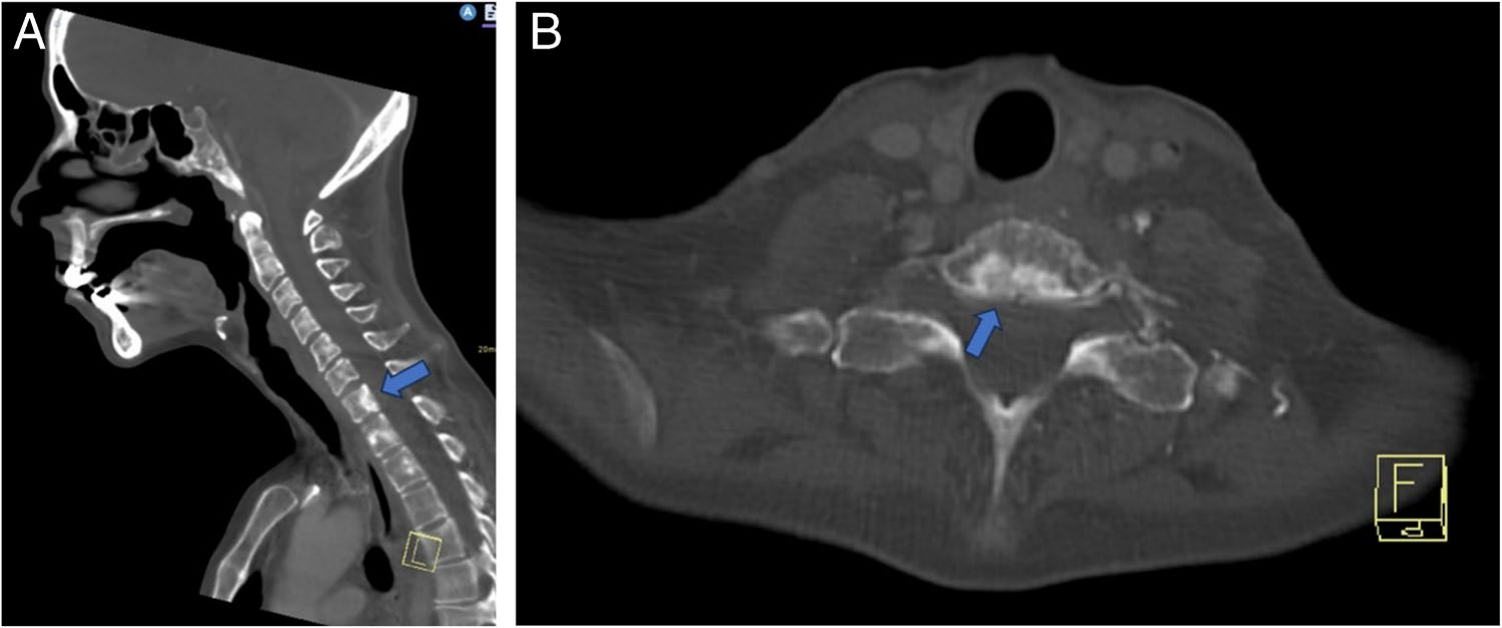
Bone algorithm sagittal midline (**A**) and axial (**B**; at the T1 level) images from contrast-enhanced neck CT demonstrate nodular hyperdensities in multiple vertebral bodies, most conspicuous in the T1 vertebral body (arrows)

**Fig. 4:**
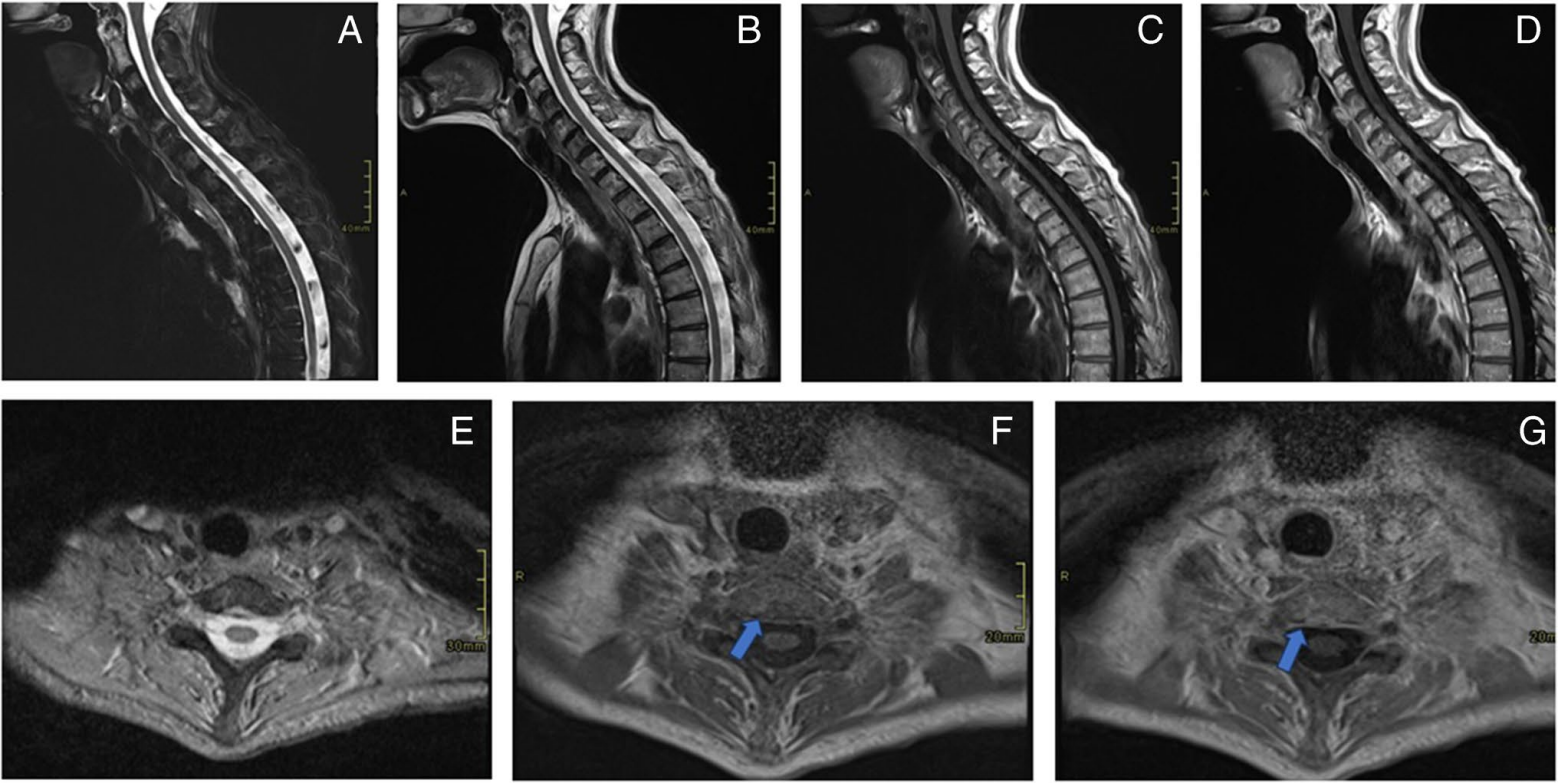
Sagittal midline short tau inversion recovery (STIR) image (**A**), T2 image (**B**), T1 precontrast (**C**) and postcontrast (**D**) images, and axial T2-weighted image at the T1 level (**E**) show mild nonspecific vertebral body marrow signal heterogeneity. Axial T1 precontrast (**F**) and postcontrast (**G**) images show T1 hypointensity and enhancement (arrows) in the region corresponding to the enhancement seen on previous neck CT images (see Fig. 1)

A 61-year-old woman with a history of nasopharyngeal carcinoma previously treated with radiation presented to the emergency department with hypotension, fever, and increased sputum production. Initial CT imaging of the chest, abdomen, and pelvis performed in the emergency department was significant only for small bilateral pleural effusions. The patient was placed on empiric antibiotics (piperacillin-tazobactam) due to clinical concern for aspiration and was admitted to a general medicine service. Two days after admission, blood cultures grew *Strep intermedius* and the patient reports neck pain and swelling; a contrast-enhanced neck CT was obtained and demonstrated an epidural abscess centered at the C1–C2 level (Fig. 1), as well as hyperdense foci in the posterior central aspects of multiple lower cervical and upper thoracic vertebral bodies, suggestive of osseous metastases (Fig. 2). The patient subsequently underwent MRI of the cervical spine without and with contrast 6 days after admission; these images showed the extent of the epidural abscess (Fig. 3) as well as nonspecific patchy mild T1 hypointensity and questionable enhancement corresponding to the hyperdense vertebral body foci seen on the CT scan (Fig. 4).

**Fig. 5:**
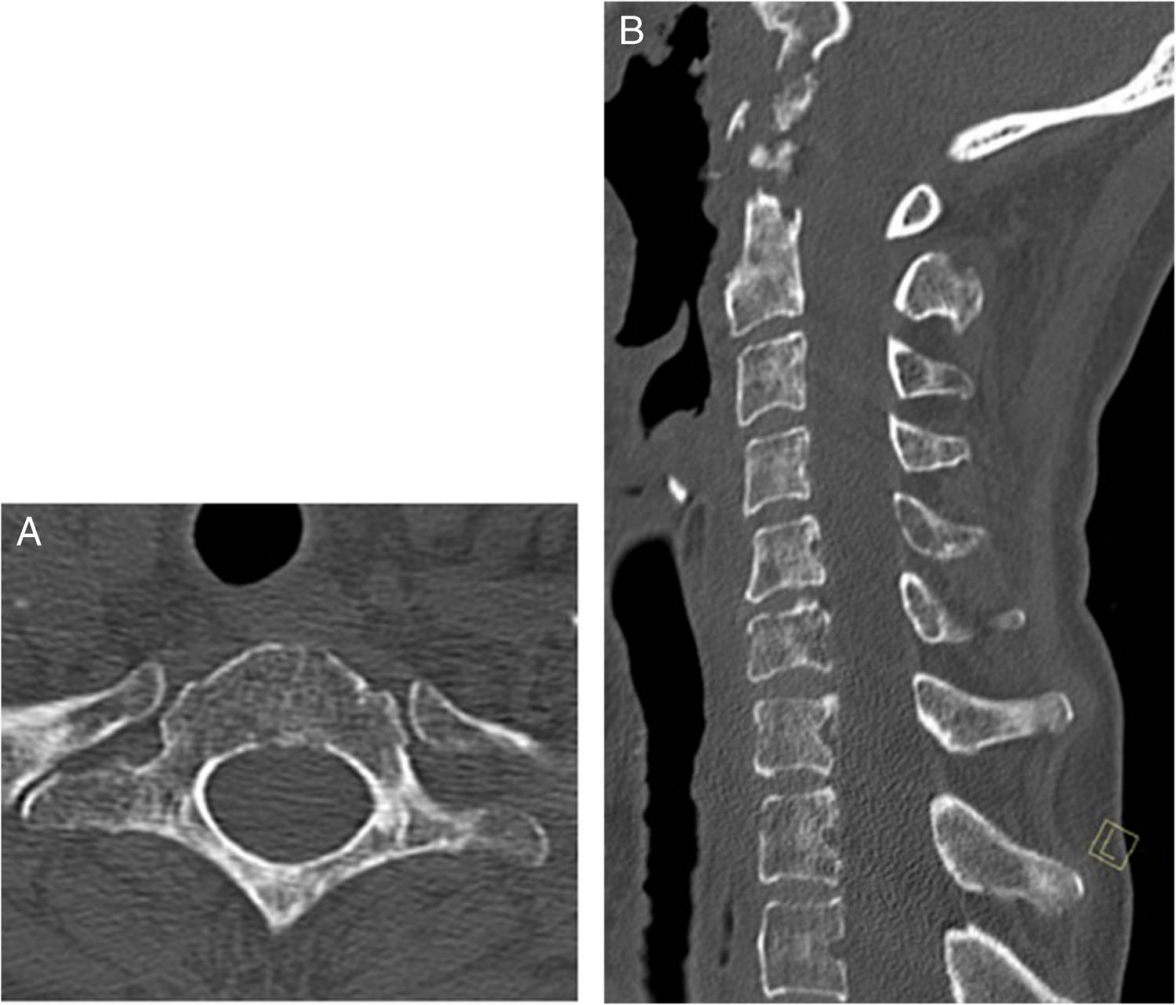
Axial (**A**; at T1 level) and sagittal midline (**B**). Noncontrast CT images demonstrate diffuse marrow signal heterogeneity. No focal sclerotic lesions are seen corresponding to the abnormalities seen on previous contrast-enhanced neck CT images (see Fig. 1)

**Fig. 6:**
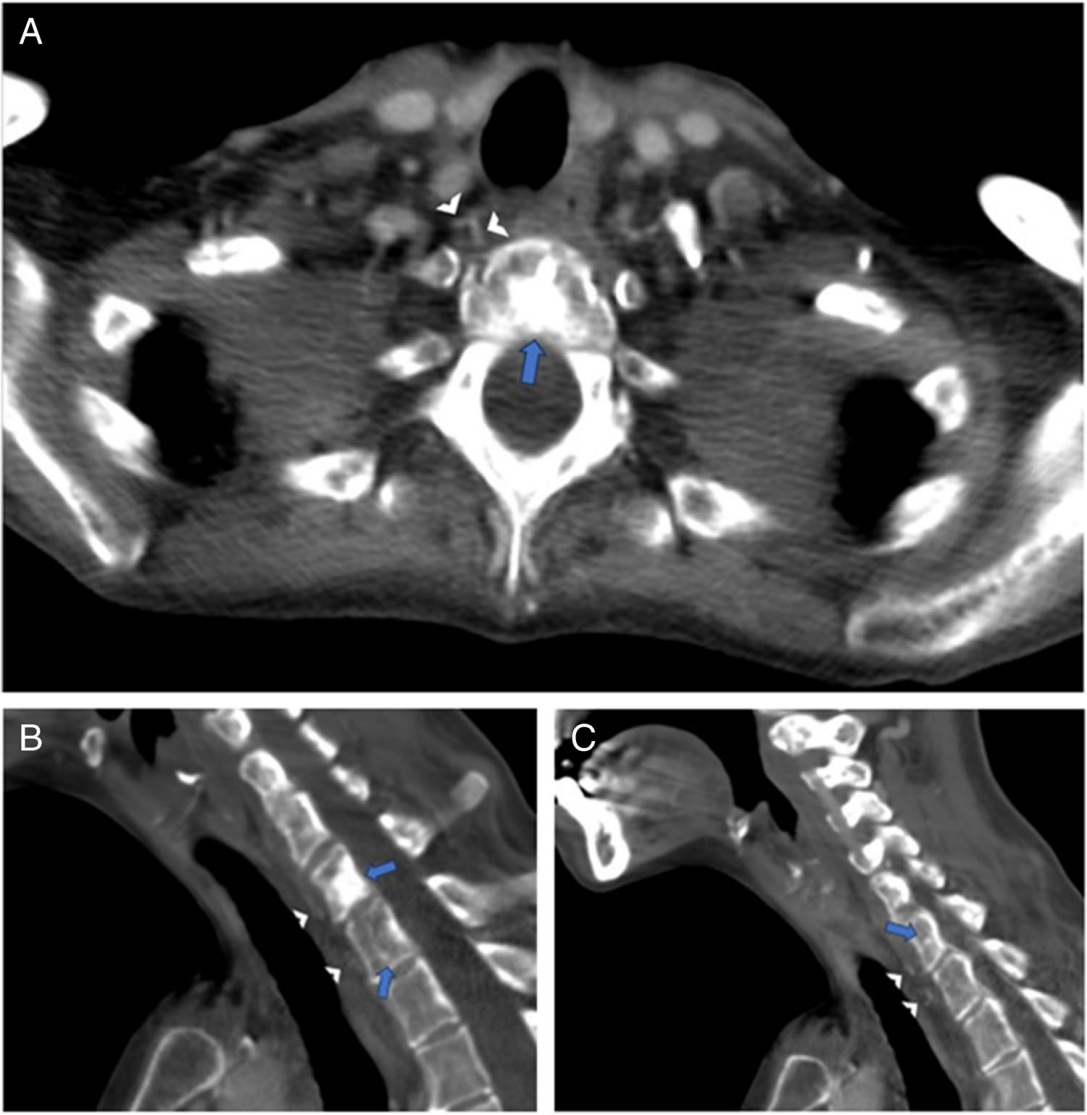
Axial (**A**) and sagittal (**B**, **C**) images from contrast-enhanced neck CT (the same study presented in Fig. 1) demonstrate tiny anterior paravertebral venous collaterals (arrowheads) adjacent to the hyperdense vertebral body foci (arrows)

**Fig. 7:**
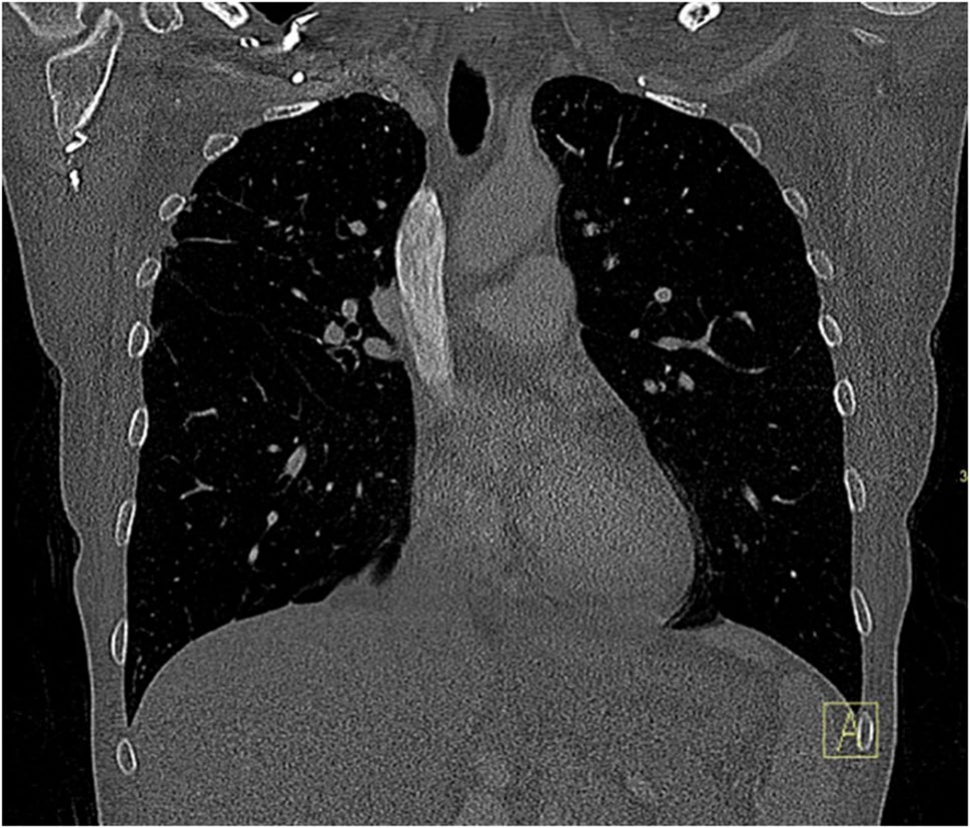
Coronal contrast-enhanced chest CT image showing a widely patent superior vena cava

A bone biopsy was then requested by the inpatient team on hospital day #7 to confirm the proposed diagnosis of metastatic vertebral body lesions. However, after multiple musculoskeletal radiologists reviewed the imaging results, a noncontrast CT scan of the cervicothoracic spine was recommended and obtained given the pattern of the vertebral body venous enhancement seen on initial imaging. The cervicothoracic spine CT obtained 8 days after admission did not demonstrate any correlate for the vertebral body abnormalities seen on previous imaging studies (Fig. 5), confirming the suspicion that the hyperdense vertebral body foci on contrast-enhanced neck CT were secondary to transient marrow enhancement. Upon careful retrospective review of the contrast-enhanced neck CT, multiple tiny paravertebral collateral vessels were seen adjacent to the foci of vertebral body enhancement (Fig. 6). A contrast-enhanced chest CT (Fig. 7) obtained at the time of presentation due to concern for aspiration was retrospectively reviewed and demonstrated no imaging features of central venous obstruction. The patient was treated medically for the epidural abscess, transitioning to ceftriaxone from piperacillin-tazobactam. A plan was established to monitor treatment response with follow-up MRI after discharge, which occurred 9 days after admission.

## Discussion

The association between vertebral body enhancement and central venous system obstruction has been well-documented. We present a case of vertebral body enhancement in the setting of upper cervical paravertebral abscess in a patient without central venous obstruction which, to our knowledge, has not been previously reported. Awareness of this entity and confirmation with noncontrast CT allowed for the avoidance of an unnecessary invasive procedure in this patient.

The existing literature suggests that elevated venous pressure in the setting of central venous obstruction (most commonly the superior vena cava and brachiocephalic veins) may result in the redirection of blood flow and development of more robust venous collaterals. Intravenous contrast agents flow through these collateral pathways, namely the retrovertebral venous plexus and the basivertebral and intraosseous vertebral veins, resulting in vertebral body enhancement [6, 8, 9].

Most previous studies have focused on the association between vertebral body enhancement and central venous obstruction; however, the patient described in this case had no imaging evidence of central venous obstruction. This is concordant with the findings from a study by Simeone et al. that demonstrated intravertebral enhancement among 5% of a control group of patients without central venous obstruction [10]. This entire subset of patients had paravertebral collateral vessels on CT, possibly related to rate and/or volume of injected contrast. This appearance has not otherwise been reported as a normal variant in patients without concomitant pathology which could account for the altered enhancement. In our case, we hypothesize that hyperemia and inflammation associated with the cervical epidural abscess may have contributed to relative flow diversion through paravertebral collaterals. Venous engorgement secondary to inflammation along the venous plexus can cause a mechanical obstruction of venous drainage. Such a mechanism would explain the observed intravertebral enhancement in the absence of overt central venous obstruction or more exuberant collateral vessels suggestive of overly voluminous or rapid contrast injection [10–12].

While there is no formal protocol for the work-up of possible epidural abscess at our institution, contrast-enhanced MR is often the first study performed in patients with a history of spine surgery due to its high sensitivity and contrast resolution. In cases of more nonspecific neck pain in patients without a history of surgery and where the pre-test probability for epidural abscess is somewhat lower, contrast-enhanced neck CT is usually obtained initially, sometimes followed by contrast-enhanced MR if the neck CT is nonrevealing. Noncontrast CT is not regularly performed at our institution when the primary diagnostic concern is for epidural abscess but may be used initially if there is concern for osseous etiologies (degenerative disc disease, facet arthropathy, etc.) or if there is a contraindication to contrast usage. In this case, noncontrast CT played an invaluable role, providing diagnostic clarity and preventing an unnecessary biopsy.

Osteoblastic metastases are most commonly associated with prostate cancer in elderly men and with breast cancer in women [13]. However, bone metastases are the most common distant metastases seen in patients with nasopharyngeal carcinoma, with an incidence of 6.6–20% [3, 4, 14–17]. Just over one in five cases of nasopharyngeal carcinoma osseous metastases is purely osteoblastic. An overwhelming majority occur in the axial skeleton, specifically the spine, which is the site of approximately 60% of initially noted osseous metastases [16]. A lower survival rate has been reported in patients with bone metastasis [17]. Therefore, accurately differentiating pseudolesions from metastases is important, as this distinction can affect disease staging, predictions about prognosis, and treatment approach [18, 19].

In conclusion, we report a case of intravertebral collateral enhancement mimicking osseous metastatic disease in the setting of cervical epidural abscess without co-existent central venous obstruction. Radiologists should be aware that central venous obstruction is not a necessary prerequisite for this enhancement pattern and may result from hyperemia associated with nearby infection. Awareness of this unique presentation may prevent the incorrect diagnosis of osseous metastatic disease, an oversight which could have profound negative consequences for patients.
